# The Relevance of Caseous Lymphadenitis as a Cause of Culling in Adult Sheep

**DOI:** 10.3390/ani10111962

**Published:** 2020-10-24

**Authors:** Héctor Ruiz, Luis Miguel Ferrer, Juan José Ramos, Cristina Baselga, Oihane Alzuguren, María Teresa Tejedor, Ricardo de Miguel, Delia Lacasta

**Affiliations:** 1 Animal Pathology Department, Instituto Agroalimentario de Aragón-IA2, Veterinary Faculty of Zaragoza, Universidad de Zaragoza-CITA, C/Miguel Servet 177, 50013 Zaragoza, Spain; hectorruiz353@gmail.com (H.R.); lmferrer@unizar.es (L.M.F.); jjramos@unizar.es (J.J.R.); ricardo.demiguel.moral@gmail.com (R.d.M.); 2 EXOPOL, Diagnóstico y Autovacunas, Pol. Río Gállego C/D, San Mateo de Gállego, 50840 Zaragoza, Spain; crbaselga@exopol.com (C.B.); oalzuguren@exopol.com (O.A.); 3 Anatomy, Embryology and Animal Genetics Department, CIBER CV (Universidad de Zaragoza-IIS), 50009 Zaragoza, Spain; ttejedor@unizar.es

**Keywords:** caseous lymphadenitis, culling, sheep, wasting disease

## Abstract

**Simple Summary:**

Caseous lymphadenitis is a widespread disease, which has been noticed in most sheep farming countries, causing important economic losses. This disease produces emaciation and weakness in the animals and has to be considered in the differential diagnosis of the so-called ‘’thin ewe syndrome’’, especially when sheep are affected by the visceral presentation of the disease. The present study analysed the prevalence of the disease in the Ebro valley area in Spain and its implication as a cause of culling in adult sheep.

**Abstract:**

Four hundred and ninety-eight culled sheep received at the Ruminant Clinical Service of the Veterinary Faculty of Zaragoza, Spain, were examined in life and after humanitarian sacrifice in order to reach the final diagnosis of the cause of culling and to evaluate the presence of caseous lymphadenitis (CLA) lesions. One hundred and forty-seven of the 498 studied animals (29.52%) showed CLA compatible lesions that were subsequently confirmed by *Corynebacterium pseudotuberculosis* isolation. One hundred and seven of the 147 CLA affected animals presenting the visceral clinical form of the disease (72.79%), while only 32 animals were affected by the superficial form (21.77%). In addition, eight animals were found to be affected in both the visceral and the superficial presentations (5.44%). Eighty-four of the 147 CLA-affected animals (57.14%) did not show any concurrent disease, considering, in this case, CLA the main cause of culling (84/498: 16.87%). In the superficial presentation, the retropharyngeal lymph node, as a sole lesion, was the most frequently affected (13/32: 40.63%). Further, in the visceral form of the disease, 85.06% of the affected animals had the lesions located in the respiratory system (91/107: 85.06%). CLA was revealed as an important cause of culling in sheep production.

## 1. Introduction

*Corynebacterium pseudotuberculosis* is a causative agent of chronic infections in a significant number of different mammalian species, such as sheep, goats, llamas, alpacas, buffalo, cattle, horses, or even humans [1,2]. The species most commonly affected by this microorganism are sheep and goats, in which the disease is called caseous lymphadenitis (CLA) [3,4,5].

*C. pseudotuberculosis* is a facultative intracellular anaerobic, non-spore-forming, non-capsulated, non-motile Gram-positive pleomorphic bacterium [4,6], and it has two major virulence factors: a potent phospholipase-D (PLD) exotoxin and a mycolic acid-rich cell wall [4,7,8].

In sheep, caseous lymphadenitis is caused by *Corynebacterium pseudotuberculosis* biovar *ovis,* and it is a highly prevalent and economically important disease in most sheep farming countries [8] because the disease can produce a negative impact on wool, meat and milk production, on carcass and skin condemnation and on reproductive performances of flocks [5,8,9,10,11]. *C. pseudotuberculosis* infection in sheep develops in the formation of pyogranulomatous lesion once the infection has become established, which can be located in different organs [12]. These pyogranulomatous lesions consist of a thick whitish caseated material surrounded by a thick fibrous wall [13], dependent on its location and whether or not the capsule ruptures, increase their size and develop on a lamellated appearance. The lesion described as an “onion ring” presentation is mostly associated with chronic CLA. This “onion ring” presentation is brought about through a process of repeated necrosis of the lesion capsule caused by the proliferation of the pathogen agent, followed by the reformation of the capsule as a protection strategy of the organism to delimit the infection [5,12,13]. 

Depending on the location of these pyogranulomas, the disease gives rise to two distinct clinical presentations [12,14,15]. The external form of CLA, also known as cutaneous or superficial form, is characterised by the development of pyogranulomas located in the superficial lymph nodes, such as retropharyngeal, parotid, submandibular, prescapular, precrural, testicular or mammary lymph nodes, or even located in the subcutaneous tissue [5,12]. This external form usually affects young animals. The visceral clinical presentation mostly affects adult animals [8]. In this case, the pyogranulomas are commonly found in the internal lymph nodes, such as mediastinal or mesenteric lymph nodes, or even in organs, such as the lungs, liver, kidneys, brain or testes [5,12,16]. Both clinical forms can appear concomitantly in the same animal, developing a combined clinical presentation [4,17]. 

Although it is a common disease in sheep flocks, it often goes unnoticed in adult animals due to the absence of apparent clinical signs of the visceral form [4]. However, it is considered that CLA can cause a chronic wasting syndrome that many authors have designated as “thin ewe syndrome” [3,8,13]. Therefore, this disease should be included in the differential diagnosis when confronted with emaciated ewes. 

Animals affected by CLA superficial form can disseminate the pathogen microorganism to the immediate environment due to the rupture or fistulisation of the abscesses, where the microorganism is able to survive for several weeks or even months. The animals of the flock are then exposed to the pathogen in the environment, and this can be introduced in the animal through wounds on the skin or mucosae; shearing being a critical risk factor to be controlled [11,12,18,19]. More recent studies have postulated that aerosol transmission of this organism is possible because animal with pulmonary CLA lesions can release the pathogen through the exhaled air, transferring the infection to free animals within the flock [11,12,20,21]. This hypothesis is based on epidemiological observations recorded in Australia. The authors found that the seroprevalence of CLA increased rapidly in a flock where there were no superficial CLA lesions [21]. Other studies suggest that internal lesions are developed as part of a systemic infection initiated elsewhere in the body. This hypothesis has been corroborated by several experimental infections performed with *C. pseudotuberculosis* by the subcutaneous or intravenous route that has led to the development of pyogranulomatous lesions at sites distinct from the inoculation site, including lungs and mediastinal lymph nodes [22,23].

Caseous lymphadenitis has been reported in the majority of the sheep rearing areas all over the world; however, its prevalence is underestimated since only a few countries, or even regions, have conducted epidemiological studies in order to establish disease prevalence rates [5,8]. Studies performed in Australia [24,25], Canada [26], the USA [20], Brazil [17,27], Iran [19] or Egypt [13] have shown a high prevalence of the disease, ranging from 12.60% to 61.00%. In Europe, some studies have been performed [28,29,30], reporting lower prevalence (0–6.4%), with Spain being the country that has shown the highest rates [29]. The most recent epidemiological investigations have been performed in Iran [19], Egypt [13], the Falkland Islands [31] and Brazil [27]. 

In the present work, the prevalence of CLA in adult culled sheep was analysed, trying to discern when CLA was the final cause of culling and when it appeared as a concomitant disease associated with other disorders.

## 2. Materials and Methods 

Following the recommendations of the Ethics Committee for Animal Experiments from the University of Zaragoza, the care and use of animals were performed accordingly with the Spanish Policy for Animal Protection RD53/2013, which meets the European Union Directive 2010/63 on the protection of animals used for experimental and other scientific purposes.

In the present study, 498 culled sheep received at the Ruminant Clinical Service of the Veterinary Faculty of Zaragoza, Spain, during the years 2017, 2018 and 2019, were analysed in detail, both in life and post mortem, in order to reach the final diagnosis of the cause of culling. 

### 2.1. Studied Farms

All animals came from six different meat sheep farms located in the Ebro valley, the Faculty’s area of influence. The production system in five of the farms was semi-intensive, keeping the animals grazing throughout the year and being housed at the end of gestation (one month) and during lactation (45 days). All these farms raised the local meat breed Rasa Aragonesa. The remaining farms were managed with an intensive production system, keeping the animals indoors through all the year. In this farm, Salz and INRA 401, both prolific synthetic breeds intended for meat production, were raised. 

### 2.2. Animal Management

All the received sheep were subjected to a rigorous clinical examination with particular attention paid to the respiratory system. All the data were collected in an individual clinical file. The age of the animals was determined according to the observed dentition. The preliminary differential diagnosis was established based on clinical signs. The superficial presentation of CLA in live animals was analysed by careful observation and palpation of the animal, thus appreciating compatible masses. Subsequently, the necessary ancillary tests (haematology, ultrasonography, thermography, computed tomography, etc.) to reach a final diagnosis were performed. Finally, all the animals were humanely sacrificed to conclude with the post mortem study. The lesions observed at necropsy and a presumptive diagnosis as the cause of culling were recorded on a pathology file. Finally, the samples were taken from the affected organs for microbiological, molecular and histopathological analysis. Visceral presentation of CLA was recorded at the post mortem study. All macroscopic lesions compatible with CLA were sampled with a sterile swap and kept with culture medium until their analysis in the laboratory for isolation of the causative agent. 

### 2.3. Ancillary Tests

Samples of blood with EDTA collected at the arrival of the animals were subjected to a haematological study, using an automatic haematological counter Vet-ABC (DIVASA-FARMAVIC S.A., Barcelona, Spain). Measured parameters were RBC (red blood cell count), PCV (packed cell volume), MCV (mean corpuscular volume) and leucocytes. In addition, microscopic evaluation of blood smears stained with quick panoptic was performed in order to perform a leucocyte formula.

As required, different diagnostic imaging techniques were performed. The devices used were: a thermographic camera: FLIR E63900, T198547 (FLIR Systems AB, Täby, Sweeden), a portable ultrasound machine: VET EICKCMEYER Magic 5000 3.5–5 MHz (Eickemeyer, Tuttlingen, Germany) and a computed tomography: 2-slice CT scanner (BRIVO CT385, General Electric Healthcare, Bengaluru, Karnataka, India).

### 2.4. Microbiological Analysis

The collected samples were refrigerated and brought in less than 24 h to the laboratory. There, the microbiological samples were surface plated onto blood agar (tryptic soy agar containing 5% sheep red blood cells) (BA) plates (Oxoid PB 5039A) and incubated in aerobic and microaerobic conditions (5–12% CO_2_) for 48 h at 37 °C. The identification of isolates was carried out by matrix-assisted laser desorption/ionisation time of flight (MALDI-TOF, Bruker Daltonics, Bremen, Germany). 

### 2.5. Statistical Analysis 

Subsequently, all the data were recorded in computer programs, such as Microsoft Office Excel 2010 and IBM SPSS statistics version 26 (2019) software (IBM, Armonk, NY, USA), which allowed the statistical study of that data. Age data were presented as mean ± SD; normality of age data was assessed by Shapiro–Wilk’s test. Since the assumption of normality was discarded, comparisons of age among groups were carried out by non-parametric test (Mann–Whitney U test and Kruskal–Wallis test). For comparing percentages, a chi-square test was used. A *p*-value < 0.05 was considered as statistically significant.

## 3. Results

Although the cause of culling of all the studied animals was analysed, the present study only focused on the CLA lesions found and the relationship with other concomitant diseases. All the animals classified as CLA-positive in this study showed CLA compatible lesions that were confirmed with the isolation of *C. pseudotuberculosis* in the analysed samples.

### 3.1. Clinical Examination 

The average age of the 498 animals included in the survey was 5.91 ± 1.73, while the average age of the CLA-affected animals was 6.15 ± 1.44, and the average age of animals culled without CLA affection was 5.80 ± 1.83. No significant differences were detected between the ages of both groups (*p* = 0.179). 

Seventy-eight per cent of the affected ewes had a body condition value below two (score one to five) and a poor appearance, as well as poor wool aspect. The superficial clinical form of CLA was diagnosed by palpation in 32 animals (32/498: 6.43%). Although the visceral form showed no apparent symptoms, animals with pyogranulomatous lesions in the lung parenchyma or mediastinal lymph node displayed dry mixed dyspnoea upon auscultation without fever.

Some of the CLA-affected animals exhibited a rare clinical presentation, resulting in CLA lesion located in unusual organs or locations that caused infrequent symptoms. Thus, one ewe suffering of recurrent tympanism had an extremely large CLA pyogranulomatous lesion located in the retropharyngeal lymph node that prevented the animal from burping and ruminating normally. Or a ewe affected by neurological signs compatible with a vestibular syndrome turned out to have a CLA pyogranulomatous lesion located inside the skull, which pressed in the cerebellum and the brain, causing this syndrome.

### 3.2. Ancillary Test

Thirty-four of 147 CLA-affected animals (23.13%) had mild chronic anaemia when analysing the haematological data. No significant results were found on the leucocyte count.

Ultrasonography was able to detect clear internal masses, which appeared as hyperechoic rounded areas, pressing the parenchyma, especially when located in lung, liver and kidney. However, when the pyogranulomatous lesions were located in internal lymph nodes, ultrasonography was not useful.

**Figure 1:**
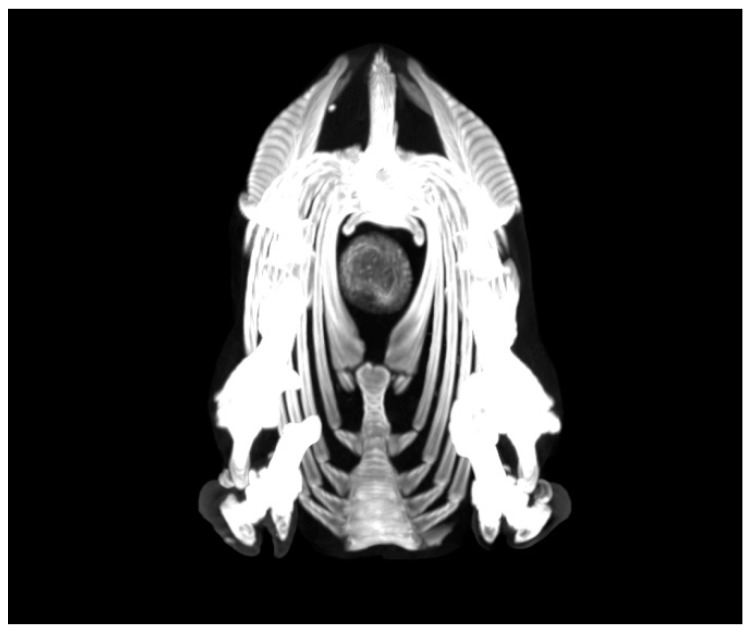
Computed tomography (CT). Axial view at the thoracic level. An enlarged mass located in the middle of the thorax in the area corresponding to the mediastinal lymph node was seen. An apparent lamellated appearance is shown with greyish layers interspersed with whitish layers. The visible notch in the lower left part of the mass is the groove through which the oesophagus ran.

Computed tomography (CT) was also a valuable technique when confronting with visceral forms. Lesions were shown by CT in different parts of the body, both organs and lymph nodes, and with a variety of aspects, from rounded masses full of material compatible with purulent abscesses to lamellated and rounded lesions (Figure 1).

### 3.3. CLA Post-Mortem Findings

As it is shown in Table 1, 147 of the 498 studied animals (29.52%) showed CLA compatible lesions that were subsequently confirmed by *C. pseudotuberculosis* isolation. One hundred and seven of the 147 CLA-affected animals presented the visceral clinical form of the disease (72.79%), while only 32 animals were affected by the superficial or cutaneous form (21.77%). In addition, eight animals were found to be affected in both the visceral and the superficial form (5.44%). 

**Figure 2:**
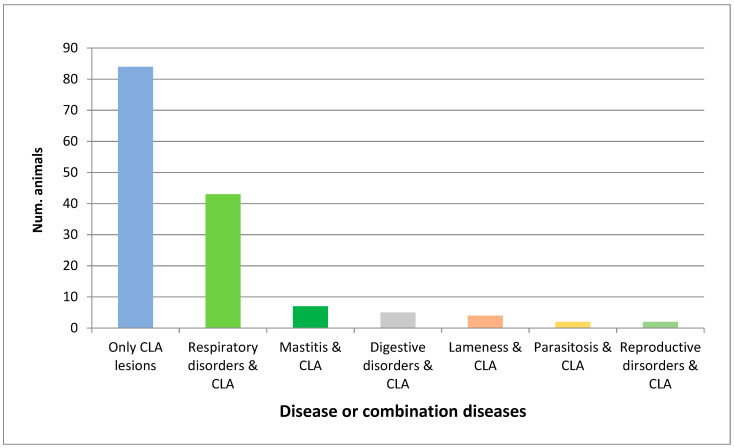
The number of culled sheep affected only by caseous lymphadenitis (CLA) or with concomitant diseases is shown. Respiratory disorders included ovine respiratory complex, pulmonary lentiviral infections, ovine pulmonary adenocarcinoma, gangrenous pneumonia and combinations of these diseases. Digestive disorders included Johne’s disease, mandibular osteomyelitis and other digestive disorders. Reproductive disorders were mainly metritis.

After analysing the main cause of culling of the studied animals, it was distinguished between those in which only CLA lesions were found and those in which these lesions appeared concomitantly with other disorders (Table 1). Eighty-four of the 147 CLA-affected animals (57.14%) only showed CLA pyogranulomatous lesions, considering this the main cause of culling (84/498: 16.87%). However, sixty-three animals (63/147: 42.86%) were concomitantly affected by other disorders. These other disorders found in combination with CLA were pulmonary lentiviral infections (18/63: 28.57%), ovine respiratory complex (17/63: 26.98%), ovine pulmonary adenocarcinoma (OPA) (5/63: 7.93%), mastitis (7/63: 11.11%), lameness (4/63: 6.35%), gangrenous pneumonia (3/63: 4.76%), mandibular osteomyelitis (2/63; 3.17%), metritis (2/63: 3.17%), Johne´s disease (2/63: 3.17%), parasitosis (2/63: 3.17%) and other digestive disorders (1/63: 1.59%). When grouping the data by systems (Figure 2), it was shown that 43 of the 63 CLA-affected animals (63.49%) had the lesions located in the respiratory system (ovine respiratory complex, gangrenous pneumonia, OPA and pulmonary lentivirosis). Some of the animals presented respiratory combined lesions.

### 3.4. Caseous Lymphadenitis Clinical Presentations 

Thirty-two of the 147 CLA-affected animals (32/147: 21.77%) had the superficial presentation of the disease, and one hundred and seven (107/147: 72.79%) were affected by the visceral form. Eight animals (8/147: 5.44%) presented a combination of both clinical forms (Table 1). 

#### 3.4.1. Superficial or Cutaneous Presentation 

The superficial form of CLA affected 6.43% (32/498) of the total of studied animals. The average age of the animals affected by the superficial presentation was 6.47 ± 1.48. In twelve of the 32 animals, CLA was the only disease diagnosed (12/32: 37.50%). When analysed the total number of animals studied, it was assumed that the cutaneous CLA clinical presentation was the main cause of culling in 2.41% (12/498) of the studied sheep (Table 1).

The retropharyngeal lymph node, as a sole lesion, was the most frequently affected (13/32: 40.63%). Further, two more animals had retropharyngeal plus prescapular and parotid lymph nodes affected, which assumed a 3.12% for each combination detected. The second lymph node more commonly affected was the mammary (10/32: 31.25%), followed by prescapular (5/32: 15.63%) and precrural (2/32: 6.25%) (Table 2). 

#### 3.4.2. Visceral Presentation

**Figure 3:**
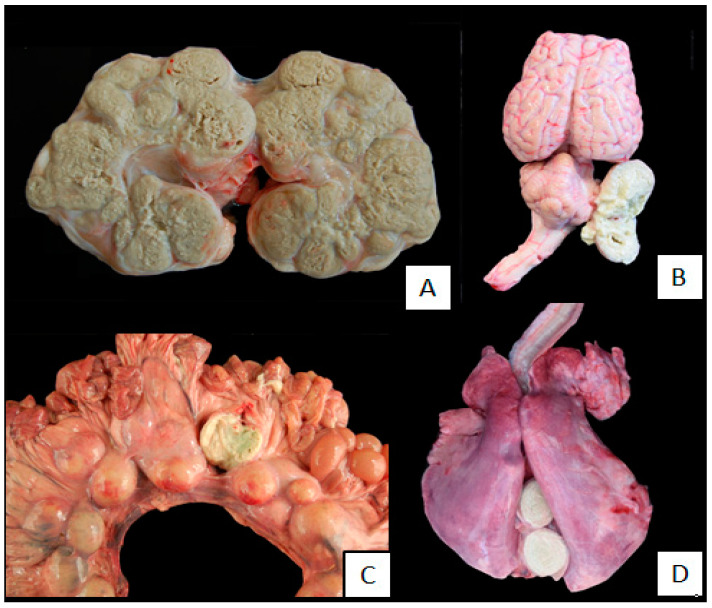
Pyogranulomatous lesions in internal organs of animals affected by caseous lymphadenitis (CLA) from which *Corynebacterium pseudotuberculosis* was isolated. (**A**). Pyogranulomatous lesions located in the kidney, almost affecting the whole parenchyma. (**B**). Pyogranulomatous lesion located next to the cerebellum. (**C**). Multiple pyogranulomatous lesions in the mesenteric lymph node, one of them opened to appreciate de caseous necrotic. (**D**). Pyogranulomatous lesion located in the mediastinal lymph node. The typical “onion ring” presentation is shown.

One hundred and seven animals of 498 were affected by the visceral presentation (107/498: 21.48%) in different locations (Figure 3) with an average age of 6.07 ± 1.45. In 65 of the 107 animals affected by the visceral form, this was the only disorder diagnosed in the animals (65/107: 60.75%), which, in relation to the total number of animals studied, represented 13.05% (65/498) (Table 1). 

Regarding the location of the lesions, 91 animals had CLA lesions located in the respiratory system (91/107: 85.06%). Within these, 36 animals had the pyogranulomatous lesion located only in the mediastinal lymph node (36/107: 33.65%), in 27, the lesion was only in the lung parenchyma (27/107: 25.23%), and 12 had a combined condition of the lung and mediastinal lymph node affected (12/107: 11.21%). Thus, 75 of the 107 animals affected by the visceral presentation had the lesion only located in the respiratory system (75/107: 70.10%). In addition, 16 animals showed lesions in other organs besides the respiratory system (16/107: 14.96%). The primary combination was the respiratory system and liver (9/107: 8.41%), followed by the combination of the respiratory tract and mesenteric lymph node (4/107: 3.74%). Finally, three animals had a combination of the respiratory system and urinary system (3/107: 2.80%) (Table 3).

Another organ frequently affected was the liver, with 18 animals having at least one pyogranulomatous lesion located in the liver (18/107: 16.82%). Five animals presented the lesion, affecting just the liver (5/107: 4.67%), while the other 13 animals had combination forms where at least two organs were affected. As it was shown, nine were affected in the respiratory system and the liver at the same time (9/107: 8.41%). The combination of liver and mesenteric lymph node was also appreciated (3/107: 2.80%). Finally, one animal was affected in the liver, kidney and brain simultaneously (1/107: 0.94%) (Table 3). 

The mesenteric lymph nodes were also affected (9/107: 8.41%). However, only one animal had the lesion located exclusively in the mesenteric lymph nodes (1/107: 0.94%). The combinations of the respiratory system with mesenteric lymph nodes (4/107: 3.74%) and liver with mesenteric lymph nodes (3/107: 2.80%) were the most frequent. In addition, a combination of mesenteric and heart was found (1/107: 0.94%). Further, six animals showed lesions in the kidney (6/107: 5.61%), but only two had the lesion limited to the kidney (2/107: 1.87%), being the most frequent combination of kidney and respiratory system (3/107: 2.80%). Finally, three animals were diagnosed with atypical forms of caseous lymphadenitis with pyogranulomatous lesions located in the mammary gland, spleen and carpus (Table 3). 

#### 3.4.3. Combined Presentations

In the present study, eight (8/498: 1.61%) animals were affected by the visceral and superficial form of caseous lymphadenitis simultaneously. The average age of these animals was 6.00 ± 1.20. Seven of them were culled with CLA disease as the only cause of culling, which, on the total number of animals included in the survey, assumed that 1.41% (7/498) was culled by a combined form of CLA.

Three of these animals had lesions located on the mediastinal lymph node and on the retropharyngeal lymph node (3/8: 37.50%) over combined forms. One of them had liver and retropharyngeal lymph node affected (1/8: 12.50%), and one had a combined condition of the liver, mediastinal lymph node and retropharyngeal lymph node (1/8: 12.50%). Other animals had affected the mediastinal lymph node, along with mesenteric and retropharyngeal lymph nodes (1/8: 12.50%). The remaining two animals had the affected prescapular and the mediastinal lymph nodes (2/8: 25.00%).

When studying the farm of origin of the animals, no significant differences were found in the percentage of CLA-affected animals (*p* = 0.252), nor in the age (*p* = 0.070). Similarly, no differences were found in the distribution of the three forms of presentation (superficial, visceral or combined) on the different farms (*p* = 0.070). 

## 4. Discussion

Caseous lymphadenitis has been reported as a highly prevalent disease in all sheep farming countries [5,8]. In the present study, 29.52% of the culled animals analysed showed CLA compatible lesions confirmed by *C. pseudotuberculosis* isolation. This percentage is much higher than that reported so far in our country. Two studies based on macroscopic lesions performed in abattoirs have described a prevalence of CLA lesions in Spain as 4.3% [28] and 6.4% [29]. However, the prevalences of lesions found in Spain are higher than in other European countries, such as France (2.3% and 5.6%), Poland (1.5%) or Italy (1.3% and 4.4%) [28,29]. In the UK, the estimated seroprevalence of CLA is 9.93% [30]. However, the percentage of affected animals in this study was lower than the results obtained in other works conducted in other sheep rearing countries. Thus, studies developed in Australia reported a CLA prevalence of 53% in 1986 in adult sheep, based on the culture of gross lesions observed at abattoirs [24]; in 1991, 61% observed gross lesions [25], while a study conducted in 2003 reported a decrease in the value by up to 26%, also based on inspection of macroscopic lesions at abattoirs [32]. Likewise, the CLA prevalence reported in the USA by observation of random lots of animals at the abattoir, in 1984, and the seroprevalence reported in Brazil, in 2011, is 43% in both cases [17,20]. In addition, in a recent study conducted in the Northeast region of Brazil, a seroprevalence of 37.46% has been reported [27]. In Canada, the prevalence rate ranges between 21 and 36% based on gross detection and subsequent isolation of *C. pseudotuberculosis* [26]. In the Falkland Islands, the prevalence is estimated close to 24% by macroscopic inspection [31], and in Malaysia, the seroprevalence is reported to be 30% [33].

In our work, it was seen that CLA was the primary cause of culling of 16.87% of the studied animals. The culling in sheep is based on the elimination of non-productive animals to improve the profitability of the farm. Although the causes of culling are many and varied, low body condition and general deterioration of the animals are the most important [34]. Almost 80% of the affected ewes in our study had a low body condition score (<2), and 23.13% presented mild anaemia, also observed by Ferrer et al. (2009) [35] in CLA-affected animals. Caseous lymphadenitis has been described as a wasting disease included in the differential diagnosis of the “thin ewe syndrome” [3,8,13]. The main and often the only clinical signs of the disease are emaciation and general deterioration of the animal [3,4,13,18]. Although the diagnosis of the superficial clinical presentation of CLA is straightforward [8,12], a differential diagnosis, including Morel´s disease, caused by *Staphylococcus aureus* subsp. *anaerobius* [36], and other diseases like tumours and mandibular osteomyelitis, should be included. However, the visceral form goes many times unnoticed. In the examined animals, it has been observed that when the respiratory system is affected, dry mixed dyspnoea could be detected, but this is a very unspecific symptom that can be confused with other diseases, such as the pulmonary form of Maedi Visna [37]. Further, depending on the location of the lesions, very varied clinical signs can be observed. Thus, in our study, one ewe showed neurological symptoms due to the location of the lesion pressing the cerebellum. A similar case was also described in goats by Leask et al. [38]. Diagnostic imaging techniques, especially ultrasonography and computed tomography, can be very useful tools to diagnose the visceral form of the disease [39,40,41]. Furthermore, in order to eradicate the disease, ELISA test can be performed, although with a sensitivity of 87% and a specificity of 98% [42], prolonging the testing period, and extending time to eradication can occur, having consequences for the economic value of the flock [43].

Caseous lymphadenitis has two clearly differentiated clinical presentations: superficial and visceral forms [12]. In the present work, visceral presentation of the disease was the most frequently found (72.79%), while only 21.77% was affected by the superficial form, and 5.44% was found to be affected by both the visceral and the superficial form. These results were supported by Pepin and Paton, who stated that the probability of developing internal lesions of CLA increased with age [8]. However, other studies have described the superficial form as the most prevalent in sheep [43] and also in goats [44]. In Iran, the authors found that 75.70% of the animals were affected by the cutaneous presentation and 24.30% by the visceral form [19]. These data are almost the opposite of those obtained in our study. However, the average age of the animals in this work was 2.92, which was lower than the average age of 5.91 shown by the animals analysed in our study, although, as explained, the age data in our study were obtained by dentition and, hence, not being entirely accurate.

In the visceral presentation, several different organs can be affected. Thus, lesions in the mediastinal or mesenteric lymph nodes, lungs, liver, kidneys, brain or testes have been described [5,12,16]. In the present study, 85.06% of the CLA-positive animals had lesions in the respiratory system. These data were in concordance with other authors who have considered the mediastinal lymph nodes and the lungs the most common visceral organs infected by *C. pseudotuberculosis* [4,12,16]. Although other authors have cited that pyogranulomatous lesions located in the chest cavity, especially in the lung, are not so frequent [31]. Likewise, Al-Gaabary et al. (2010) reported that the most frequently affected internal viscera was the liver (69.44%), while the mediastinal lymph node and lung were less commonly affected (30.56%) [13].

Regarding the cutaneous presentation, in our study, the most frequent location was the retropharyngeal lymph node, followed by the mammary lymph node. This coincided with the results obtained by Al-Gaabary et al., who found that the superficial cervical lymph nodes, in which retropharyngeal can be included, were the most frequently affected [13]. However, Zavoshti et al. found prescapular lymph node lesion as the most common superficial presentation [19].

## 5. Conclusions

Based on the prevalence studies presented here, caseous lymphadenitis has been revealed as a significant disorder of culling sheep (29.52%), being the primary cause of culling of 16.87% of the studied animals. However, this disease has been many times neglected because the visceral form that is the most frequent clinical presentation produces very unspecific clinical signs. Deterioration of the animals and the chronic weight loss associated with the disease lead to the early culling of the affected animals. Therefore, CLA is a disorder that must be included in the differential diagnosis of wasting diseases in sheep production.

## Figures and Tables

**Table 1:** Number and percentage of CLA-affected animals regarding the total number of studied animals and the total CLA-affected animals.

CLA Presentations	Num. of CLA-Affected Animals of Total Studied (498)	Percentage of Total Studied Animals	Num. of Affected Animals of Total CLA-Affected (147)	Percentage of CLA-Affected Animals
CLA	147/498	29.52%		
Visceral form	107/498	21.48%	107/147	72.79%
Superficial form	32/498	6.43%	32/147	21.77%
Combined form	8/498	1.61%	8/147	5.44%
Unique CLA lesion	84/498	16.87%	84/147	57.14%
Unique visceral presentation	65/498	13.05%	65/147	44.21%
Unique superficial presentation	12/498	2.41%	12/147	8.16%
Unique combined presentation	7/498	1.41%	7/147	4.76%
CLA + concomitant diseases	63/498	12.65%	63/147	42.86%

**Table 2:** Number and percentage of the affected superficial lymph nodes (LN).

Affected Lymph Nodes	Number	Percentage
Retropharyngeal LN	13/32	40.63%
Mammary LN	10/32	31.25%
Prescapular LN	5/32	15.63%
Precrural LN	2/32	6.25%
Prescapular + retropharyngeal LN	1/32	3.12%
Retropharyngeal + parotid LN	1/32	3.12%

**Table 3:** Number and percentage of the affected visceral lymph nodes (LN) and organs.

Affected Lymph Nodes	Number	Percentage
Mediastinal LN	36/107	33.65%
Lung parenchyma	27/107	25.23%
Mediastinal LN + lung parenchyma	12/107	11.21%
Liver + respiratory system	9/107	8.41%
Mesenteric LN + respiratory system	4/107	3.74%
Kidney + respiratory system	3/107	2.80%
Liver	5/107	4.67%
Liver + mesenteric LN	3/107	2.80%
Liver + kidney + brain	1/107	0.94%
Mesenteric LN	1/107	0.94%
Mesenteric LN + heart	1/107	0.94%
Kidney	2/107	1.87%
Atypical presentations	3/107	2.80%
